# 12,000-year-old spindle whorls and the innovation of wheeled rotational technologies

**DOI:** 10.1371/journal.pone.0312007

**Published:** 2024-11-13

**Authors:** Talia Yashuv, Leore Grosman

**Affiliations:** The Computational Archaeology Laboratory, Institute of Archaeology, The Hebrew University of Jerusalem, Jerusalem, Israel; University of Haifa, Zinman Institute of Archaeology, ISRAEL

## Abstract

‘The wheel and axle’ revolutionized human technological history by transforming linear to rotary motion and causing parts of devices to move. While its ancient origins are commonly associated with the appearance of carts during the Bronze Age, we focus on much earlier wheel-shaped find–an exceptional assemblage of over a hundred perforated pebbles from the 12,000-year-old Natufian village of Nahal Ein-Gev II, Israel. We analyze the assemblage using 3D methodologies, incorporating novel study applications to both the pebbles and their perforations and explore the functional implications. We conclude that these items could have served as spindle whorls to spin fibres. In a cumulative evolutionary trend, they manifest early phases of the development of rotational technologies by laying the mechanical principle of the wheel and axle. All in all, it reflects on the technological innovations that played an important part in the Neolithization processes of the Southern Levant.

## Introduction

Circular objects with a hollowed centre connected to a bar make one of the most important inventions of all time. By causing parts of devices to move, wheels brought about inventions that have revolutionized human transportation, energy exploitation, engineering and the mechanical industry [1]. From carts, cars, potter’s wheels and power mills, oil/wine-pressers, lathes, spinning wheels and many other applications, each invention has had its distinct footprint on the history and evolution of technology [2]. At the core of it all, the importance of ‘the wheel and axle’ lies in a relatively simple rotational mechanism capable of transforming linear to rotary motion and vice versa [3].

Gordon V. Childe [4] was interested in rotation motion technologies, as he believed that the Industrial Revolution’s main innovations were extensions of earlier rotary applications. He distinguished instruments and actions of ’partial’ rotary motion from ’continued, true, complete’ rotary motion. While the former includes drilling and fibre-spinning known from prehistoric times, the latter addresses the superiority of the wheel–“a disc equipped with bearings to allow it to spin freely” (p. 194) [4]. The substrate of the rotation mechanism existed long before the wheel and axle of a vehicle, and Childe paved the way to explore a broad evolutional quest for the wheel’s antecedents.

However, while investigations usually focus on the evolution of functionally related technologies, a broad evolutional quest should also look at mechanical principles. As with other evolutionary processes, the ‘mechanical kingdom’ [5] is assembled by combinatorial steps, the evolution through recombination [6]. Its basic idea states that new technologies (i.e., inventions) do not come out of nowhere and that these arose as a "combination of other technologies" (p. 2) [7] or are “the constructive assimilation of pre-existing elements into new synthesis” (p. 11) [8]. In this process of recombination, elements that were previously unconnected are combined, or associated components are joint in new ways. Recombination forms a central concept in modern industrial systems that study innovations to evaluate potential integration, economic value and social effect [9, 10]. The concept of recombination also paved its way into the archaeological frame of thought of artifact analysis, joining other theoretical models that explain technological change [11–13].

On this occasion, we present and focus on a specific technology at a particular point in time: an extraordinary early assemblage of perforated stones from the Late Natufian site of Nahal-Ein-Gev II (NEG II), dating to 12,000 years ago. We draw the prehistoric context of the site and describe several properties of the assemblage–including the raw material, shape and modification marks. Followed by 3D computational analysis, we reconstruct these items as spindle whorls, a tool used for spinning fibres into yarn. As we delve into the properties of the NEG II spindle-whorls assemblage, we show these objects’ rotational potential as intrinsic to the mechanical properties of wheels and discuss how this sheds light upon understanding the dynamics of the rotational technologies’ innovation processes.

### The assemblage

The site of Nahal Ein-Gev II is located in the Jordan Valley, about 2 km east of the Sea of Galilee, on the banks of the Ein-Gev wadi. Excavations exposed a multi-layered sedentary village of a single cultural entity, the Late Natufian, dated to the very end of the Epipaleolithic (12k BP), just before the Neolithic [14]. Various aspects of technology, style and symbolism mark NEG II as continuing Epipaleolithic traditions but also point to the Neolithic changes to come. This mélange is reflected in the characteristics of the flint and ground stone tools, the architecture, burial customs and art objects [14–18].

**Fig 1:**
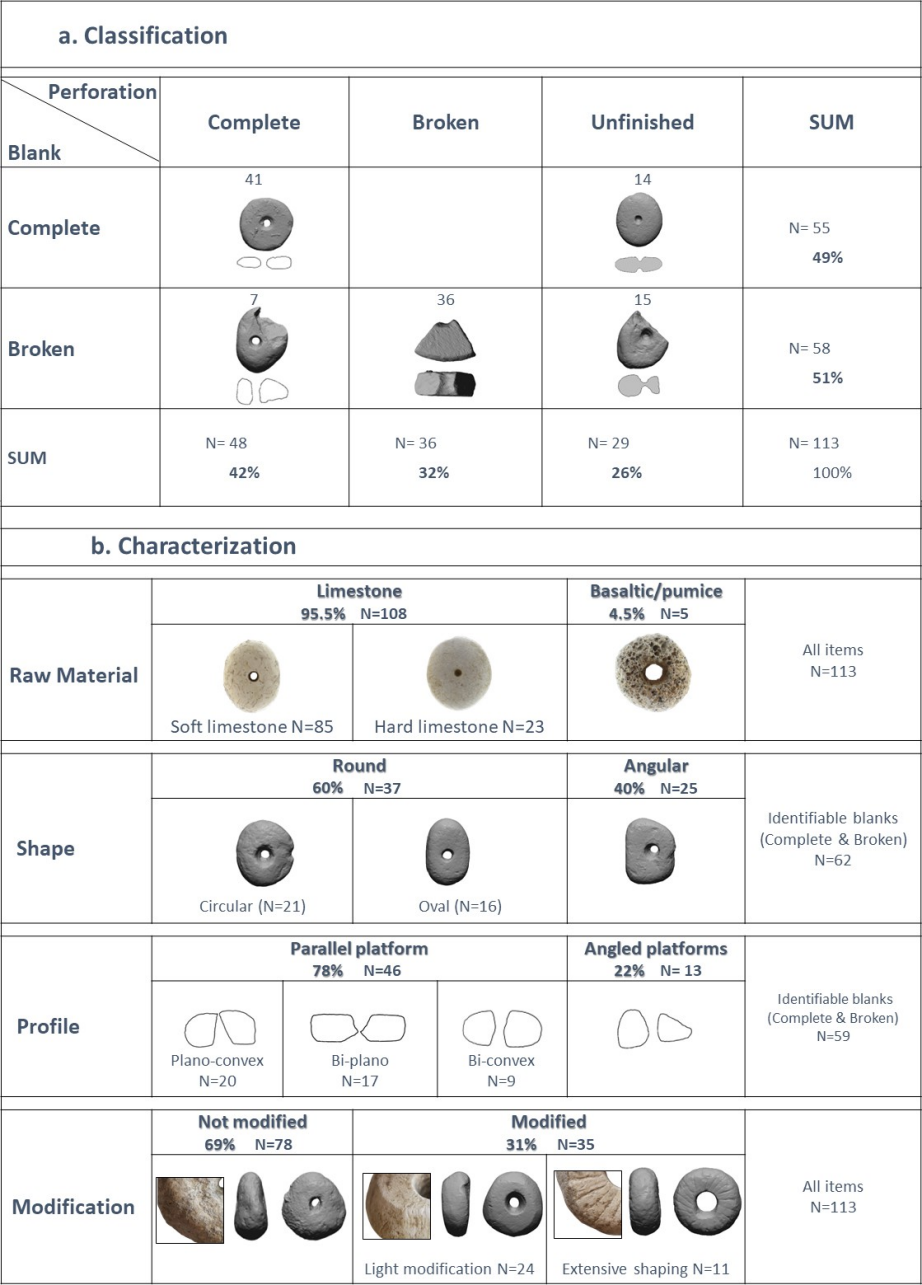
Perforated stones breakdown. (a) Classification (b) Characterization (photographed by Laurant Davin).

The excavations at NEG II yielded 113 perforated stones. Six items were retrieved from the test excavation in 1972 [19] and 107 from seasons 2010–2021. The assemblage is classified to three groups according to the state of perforation: 48 items with complete perforation (42%), 36 broken items with partial holes (32%), and 29 unfinished items with one or two drill marks (36%) (Fig 1A), which suggest the local production of the items. Preliminary analyses of the Ground Stone Tools (GST) show overall high frequency of this category (ca. 15%), being the most abundant formal tool category.

Traditional naked-eye characterization of the assemblage (Fig 1B) revealed that the raw material is dominantly limestone (95.5%), mostly of soft chalky pebbles (74%), with a few items made of basaltic minerals. Similar soft-limestone-pebbles raw material is available close to the site in a few localities: some at the Ein-Gev Wadi, a few meters away from the site, and large concentrations at the Sea of Galilea lakeshore, which was approximately at a similar distance as today from the site (about 1.5 km away). Moreover, the shape variability of the artifacts seems to reflect that of the natural pebbles. Of the identifiable shapes of the pebbles, 60% are round and symmetrical in form, while the rest are represented by more angular and irregular shapes. The majority of the pebbles (78%) have two parallel platforms/faces that form three different profile: plano-convex, bi-plano/flat, and bi-convex.

Respectively, we find that the collected pebbles were not standardly modified. Extensive shaping was applied only to 11 pebbles (10%), mostly producing round smoothed shapes and flattened profiles. Random modification signs are observed on 21% of the pebbles (N = 24, 21%), including flaking and pecking percussion marks, along with abrasive rounding and faceting marks, and the rest (69%) have no clear modification marks (N = 78,).

## 3D methodology

The morphology of the perforated stones was examined from two perspectives: the complete shape of the stones and the perforations themselves. The shape of the perforated stones was captured using a structure-light scanner (Polymetric–PT-M Scanner), which produced high-resolution 3D models [20, 21]. From the 3D digital mesh of the complete artifacts, a designed algorithm extracted 3D models only of the perforations (all 3D models available at https://zenodo.org/records/11124677). The digital-based methods available with the *Artifact3-D* software (available at https://sourceforge.net/projects/artifact3-d/) [22] were used to automatically and manually extract metric parameters.

### Complete shape analysis

**Fig 2:**
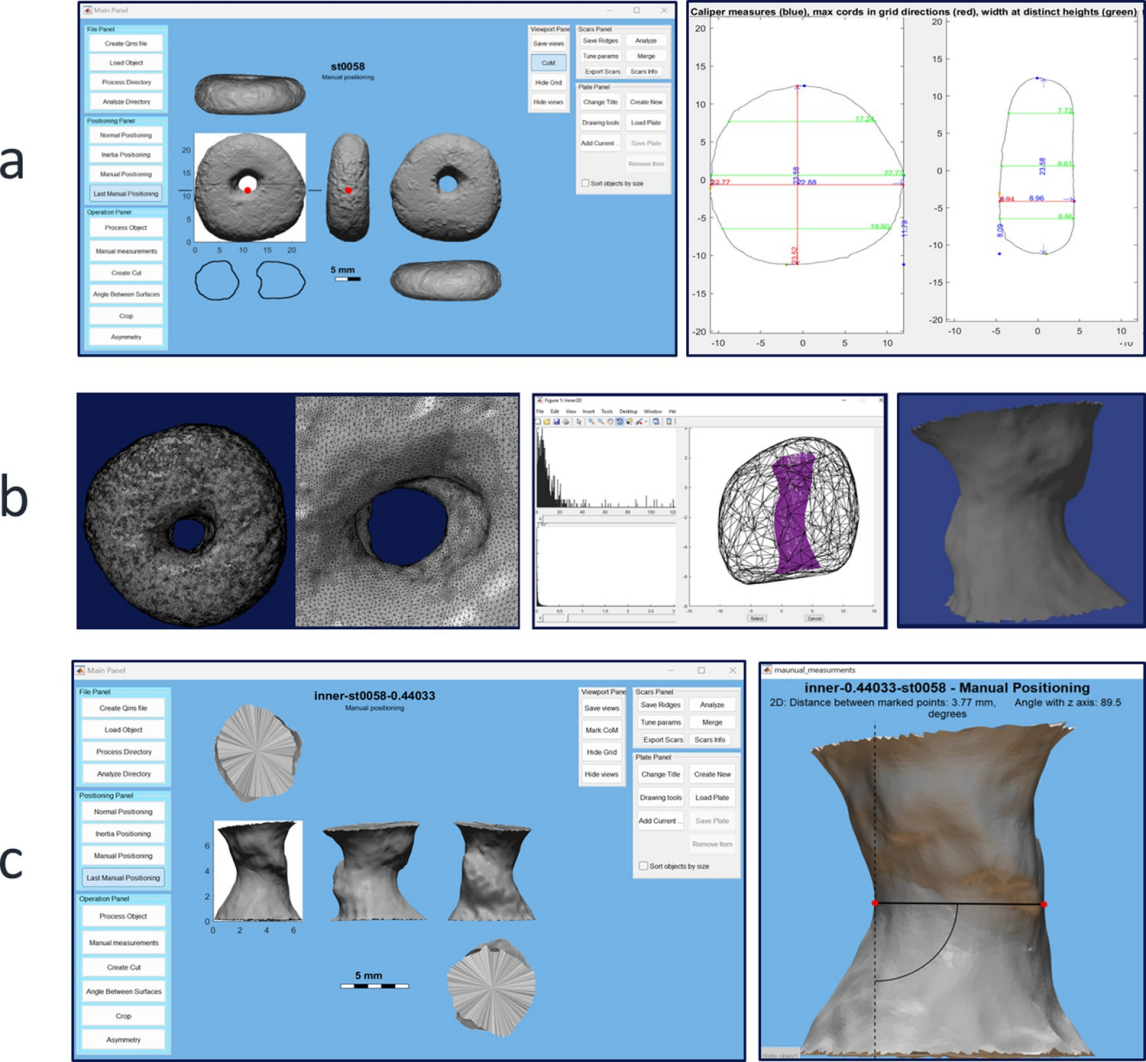
3D analysis of the perforated pebbles and the perforations. a) An example of the analysis procedure for perforated stones using the Artifact3-D software of the Computational Archaeological Laboratory, Institute of Archaeology at the Hebrew University of Jerusalem. b) The designed algorithm for producing a 3D negative of the perforations. Left-to-Right: The 3D mesh of the object, including its detailed scanned hole; The algorithm calculates the convex hull of the artifact’s mesh (black lines) and marks the most distanced points within a defined interval (in purple) to produce a negative 3D model of the perforation. c) The model of the perforation is analyzed using Artifact3-D software.

The 3D models of the complete artifacts were automatically positioned based on the direction of the normal vectors [23]. For broken items, the correct positioning followed consistent movement applied digitally to all axis. Based on the standard positioning, metric parameters were automatically extracted, including maximum length, width and thickness, volume and centre of mass (Fig 2A).

### Perforation shape analysis

The perforations were studied with a designed algorithm that transformed the geometrical mesh of the artifacts’ internal scanned surface into separate 3D models. The algorithm extracted the most distanced points from the convex hull of the artifact’s mesh, which marks the relevant inner surface of the hollowed space. Next, it exported this mesh as an independent 3D model, producing a negative model of the perforations (Fig 2B). The *findInner* algorithm, designed for the current research purposes, is nevertheless applicable to various perforated artifacts (available within the MATLAB code of *Artifact3-D* software, https://sourceforge.net/projects/artifact3-d/).

The full 360° view of the perforations allowed us to describe and measure attributes unattainable otherwise. Descriptive parameters of the perforation’s shape included its drilling shape (bi-conic, flat), the meeting point between opposite drills (central, edge, offset), the apertures’ shape (circle, round, asymmetric, oval), and the degree of similarity in the shape of the two artifact’s apertures (similar, identical, large & small).

Moreover, accurate metric parameters were taken (Fig 2C). The 3D models of the perforations were automatically positioned based on the eigenvectors of the inertia tensor [23]. The minimum width of the hole was manually measured in 3D, visually selecting the thinnest spot. An additional measurement was taken in the 2D section to ascertain the perforation’s thinnest location.

## Results

### Complete shape analysis

**Fig 3:**
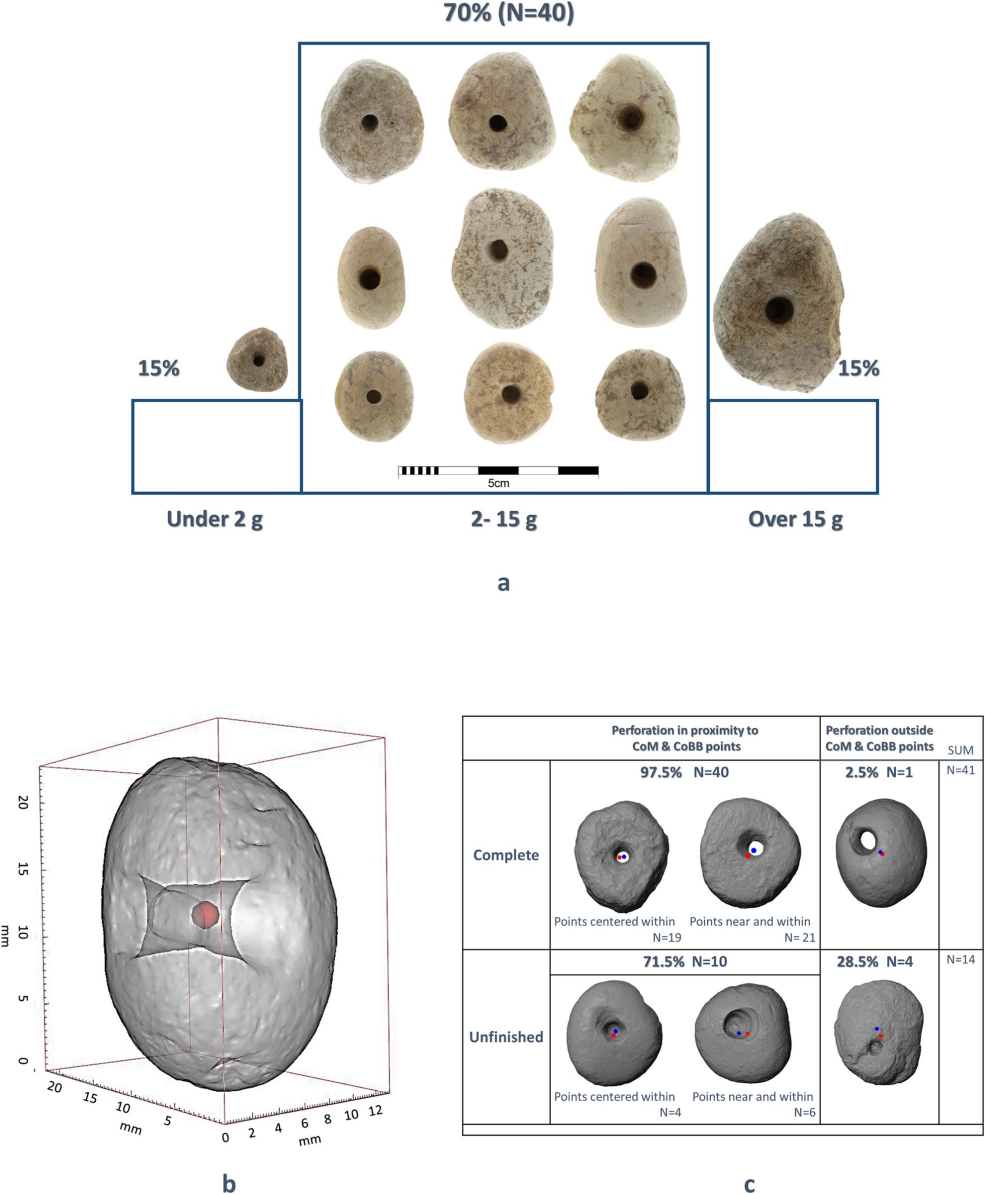
Complete shape 3D analysis of the perforated pebbles. (a) Selected artifacts that present the main weight range (photographed by Laurant Davin) (b) The binding box of a perforated pebble, with the millimetric scale and the centre of mass marked in red. (c) The location of the perforation in relation to the item’s Centre of Mass (red) and Centre of Binding Box (blue).

The artifacts are all light weighted in the range of 1–34 gr, with an average of 9 gr. ± 7 gr. The majority of the artifacts (70%) fall in the range of 2–15 gr (Fig 3A). The item’s centre (the binding box, CoBB) and the centre of mass (CoM [22]) points are close, with an average distance of 0.8 mm ± 0.3 mm between one another (Fig 3B). The ratio between the maximum length and maximum width, calculated based on the 3D binding box properties, shows a standard measure of 1.22 ± 0.14. These two parameters complement the information regarding the pebbles (Fig 1B), which are mostly round or slightly oval, with an even distribution of mass.

The location of the perforation is always positioned at the centre of the pebble. In 97.5% of the complete perforated items (N = 41), the perforation is located within or near the CoBB and the CoM points. Only a single perforation was completed farther from the item’s centre (Fig 3C). Interestingly, four of the unfinished items (28.5%) have drill marks outside the centre and were perhaps discarded due to this inappropriate offset location of the hole.

### Perforation analysis

**Fig 4:**
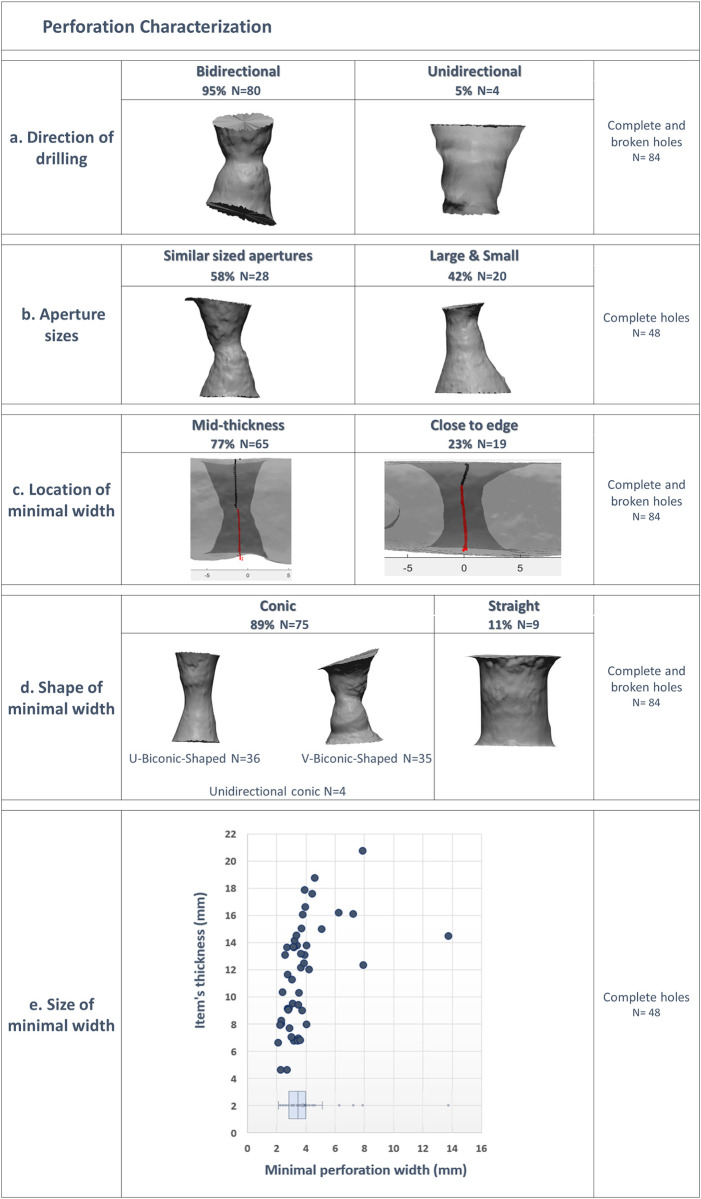
Perforation analysis. (a) direction of drilling, (b) aperture sizes, (c) location of minimal width, (d) shape of minimal width, (e) size of minimal width.

Another protocol was identified in the drilling procedure. In 95% (N = 81) of the complete and broken items, the holes are drilled bi-directionally (from two opposite directions) (Fig 4A). After trying drilling similar soft limestone pebbles picked near the site, we found that drilling from one direction all through the pebble is easily attainable, yet only 5% of the assemblage clearly shows this perforating method. The fact that one aperture is larger than the other in many items (40%, (Fig 4B) signifies that the larger one was initially drilled to a deeper depth, as the depth of the drilling affects the size of the aperture [24]. Still, the meeting point of the two opposite drills, meaning, the location of the minimal width of the perforation, was preferably the centre point, as it is usually located at the pebble’s mid-thickness (77%, (Fig 4C).

Moreover, the meeting point was only slightly widened, as shown by the dominance of biconical shapes. Only in 9 items (11%) was the minimal point of the bi-directional drilling widened to form a straight profile. Among 89% of the complete and broken items, the initial shape of the perforation was maintained, either as a sharp V-biconic-shaped (41%), a soft U-biconic-shaped (42%) or unidirectional conic-shape (5%) that reflects a minimal widening of the meeting point between the opposite drills (Fig 4D). Measurements of the minimal width (in 3D and 2D) were found to be in a limited interquartile range of only 3–4 mm, 3.6 ± 1.3 on average (min = 2.1 mm; max = 8 mm, one extreme value of 13.7 mm). These confirm a standardized measure not affected by the perforation’s shape or the pebble’s thickness (Fig 4E).

Summing up the results, the assemblage seems to reflect a natural variability in pebble shapes with little intentional modification. However, the finds point to a selective collection of preferably soft limestone pebbles with specific attributes, possibly from a close-by location. The essential sought-after qualities were light stones, generally round-oval in shape, with two parallel platforms and an even distribution of mass. The perforation was notably located at the item’s centre, which is also its mass centre. It was drilled bi-directionally, and the meeting point between the two drills was preferably at the item’s mid-thickness. The shape of the bi-directional drilling was also kept, preserving the un-uniform rotational qualities of the drilling process. The minimal hole opening was not widened much and was found to be of a standardized width—3 to 4 mm.

### Spindle whorls?

**Fig 5:**
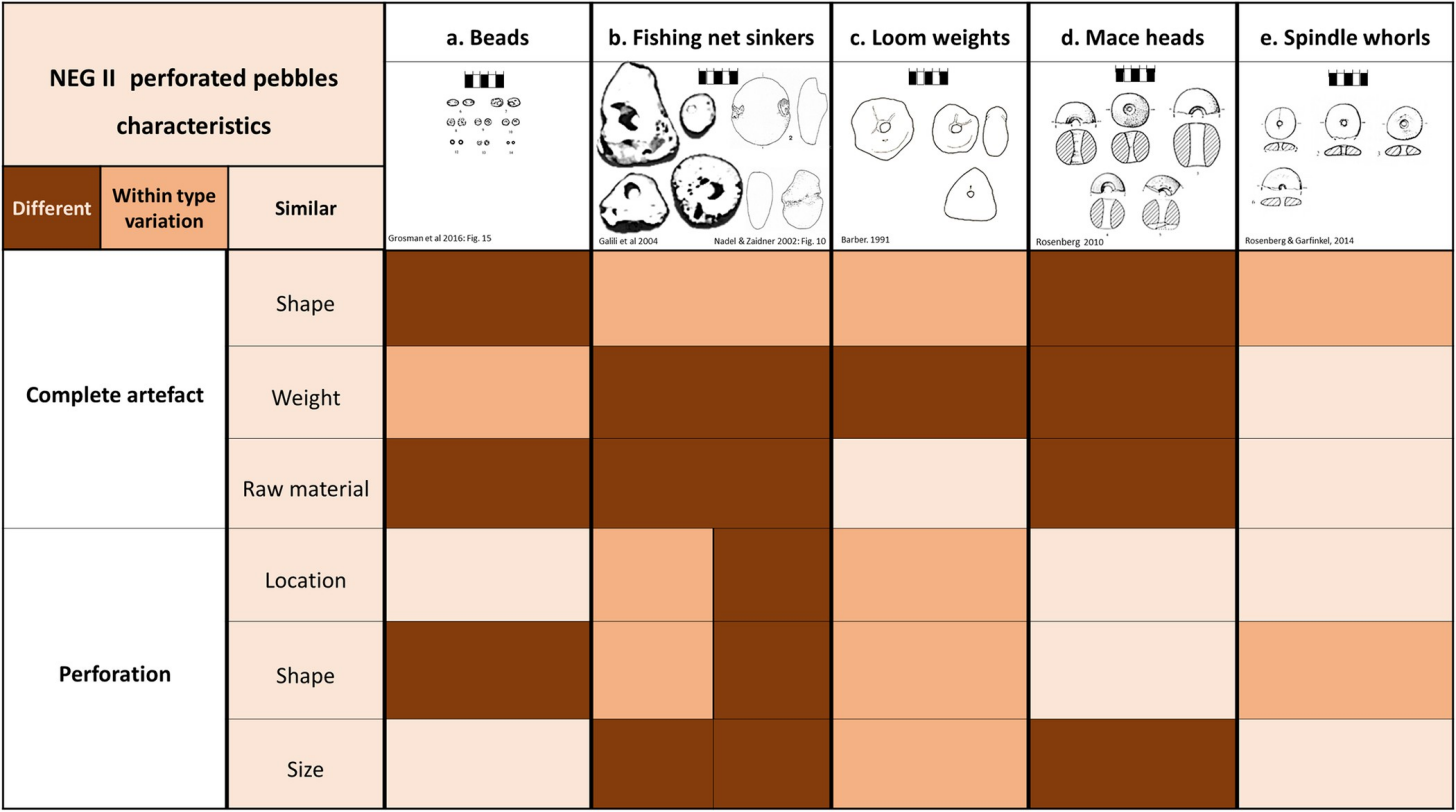
NEG’s perforated stones characteristics compared to functional interpretations. (a) beads, (b) fishing weights, (c) loom weights, (d) mace heads, (e) spindle whorls.

In this section, we wish to explore the function of the perforated pebbles from NEG II, given the suggested interpretations in the literature. Early perforated stones are commonly understood as beads, loom weights, fishing nets, mace heads and spindle whorls [25–27]. We extracted the function-related features of each tool type to confirm or negate our hypothesis that the NEG II tools functioned as spindle whorls (Fig 5).

#### Beads? (Fig 5A)

Typically, beads are distinguished by their extensive modification, highly regular shapes, tiny size and light weight. The highly homogenized disc beads retrieved in NEG II [14] are noticeably different from the perforated stones. Even the first six perforated pebbles found during the 1970’s test excavation stood out from the Natufian ornamental tradition and evoked other explanations [19]. Important to note, that some of the lightest perforated pebbles that do not exceed 2 grams may have been beads, but this cannot explain the complete assemblage, only its smallest specimens. Although there are occasions of the use of soft raw materials for the production of beads, the use of chalky limestone is not common. Natufian beads are usually produced from sea shells, fossils, and exotic minerals [28]. Last, other than the shared attribute of having small central perforations, beads are mostly shaped after the initial drilling as part of the effort to modify their aesthetic form [29].

#### Fishing weights? (Fig 5B)

When considering fishing tools, lightweight items could theoretically suit cast nets rather than a vertical net [30], with the support of having ethnographic and modern cast-net counterparts weighting about 9 grams with a 1–3 mm hole [31, 32]. However, there is no archaeological evidence for the use of this technique and associated implements in prehistoric times, with the only available perforated fishing weights from Atlit Yam being much heavier [32, 33]. In contrast, many notched and incised weights are understood to function as fishing net sinkers at water-close sites, particularly from the Epipaleolithic and the Neolithic Jordan Valley (e.g. Ohalo II, JRD, Beisamoun [34], and Sha’ar Hagolan [35]). Most of those fishing weights were made of hard minerals, possibly due to their heavy mass and long durability. Identical items were recovered from NEG II, all but one made of hard minerals (Dubreuil, personal communication). The fact that limestone and surely chalk are considered relatively soft minerals that are more fragile and may disintegrate in water [36] makes the perforated pebbles assemblage less prone to be used as aquatic gear.

#### Loom weights? (Fig 5C)

The warp-weighted loom is considered to have been widely used only from the Bronze Age onward, and in earlier weaving frames like the ground loom, no use was made of perforated weights [37, 38]. It is possible that, in-between developments of looms may have used weights even if just for holding the strings [39], but no supporting evidence for this has been found to date. Nevertheless, a large assemblage of ‘perforated pebbles’ (Type G2) from Sha’ar Hagolan was suggested as net or loom weights [35]. However, while the natural pebbles are similar in shape, as is the perforation size, these are almost always edge-perforated, contrary to the NEG II items, and present a higher weight range that match that of loom weights from later periods [40]. Moreover, suspension stone tools/weights will best perform by having uneven shapes, with the centre of mass located at their base and the perforations at the farther end [41].

#### Spindle whorls? (Fig 5E)

The persisting choice of perforating pebbles at their centre of mass suggests that the composite tool is balanced and use of stick rather than string [35]. While drilling flywheels are noticeably heavier [42], and mace heads are mostly piriform in shape and weigh more than 150 g (Fig 5D) [43, 44], spindle whorls that act as flywheels to enhance rotation momentum when spinning fibres to yarn, fit best.

Spindle whorls must have a central perforation, also demonstrated in experiments [38, 40, 45]. Commonly, they are standardly rounded, resembling the more carefully modified NEG II perforated pebbles. Most importantly, the central location of the centre of mass and perforations, even among the non-standardized, natural, pebbles (see Figs 1 and 3), supports the notion that this characteristic relates to the functional requirements of the objects, most probably for balancing the items.

During the fibre spinning process, the whorls’ weight affects the thickness of the produced yarn. The weight of these whorls usually range between 15 and 35 g, with the lightest, 2 g, used to make extra fine yarn, and the heaviest, 50 g, used to spin thick strings [46–48]. Weights that are too heavy would tear the delicate fibres in the spinning process aimed at making durable threads.

The NEG II items are (a) bi-directionally drilled; (b) have a minimal meeting point between the opposing drills that is preferably centered at their mid-thickness; (c) have a relatively constant minimal width. These specific characteristics were probably purposely chosen to help with the tool’s balance. Interesting to add, that in spindle whorls, the perforation shape is related to the production technology. In general, bi-directional drilling in stones or pottery sherds produces biconical shapes, while a stick inserted into plastic clay results in a straight shape [49]. Either way, the perforation must keep the balance by having a symmetrical shape [50]. This choice is strengthened as the softness of the raw material makes it easy to perforate in a single drilling direction, as well as straightening the perforations shape.

**Fig 6:**
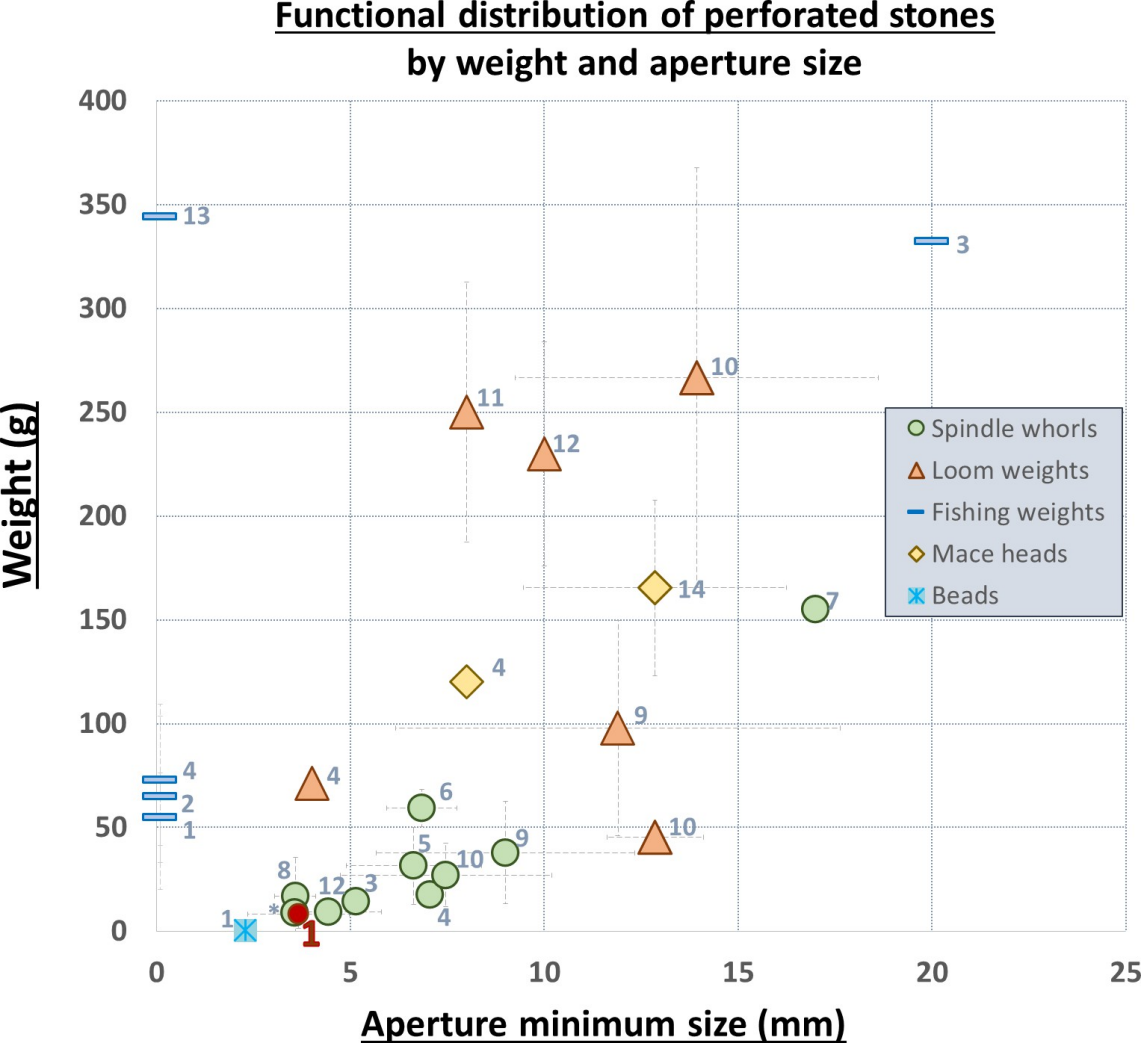
Distribution graph of perforated tools according to their published weight and minimum aperture size. Numbers = Sites & References.

In the literature, the hole size is one of the leading characteristics differentiating perforated- items functions [42, 51]. Liu [52], who surveyed ethnographic accounts of spindle whorls, reported the smallest perforation width to be 2–4 mm, the most common width being 7–8 mm and the largest—10 mm. Archaeological finds show the same width range, even for rare wooden spindles [48, 53, 54]. We compared the available information on various perforated items by hole size and weight, and the resulting graph (Fig 6) shows how the NEG II assemblage falls within the lower range amid other spindle-whorl assemblages from all periods, distinct from all the other types of perforated specimens.

**Table:** 

*1) NEG II*	*5) Gilat* [50]	*9) Kadesh Barnea* [55]	*13) Ohalo* [56]
*2) Beisamoun* [57]	*6) Qina Cave* [53]	*10) Tell Abu Kharaz* [58]	*14) Mace heads from Israel* [59]
*3) Atlit Yam* [32,60]	*7) Ashalim Cave* [53]	*11) Tell Rehov* [61]	* UP perforation size is reported [62]*; weight is estimated based on the reported dimensions*
*4) Sha’ar Hagolan* [35, 43]	*8) Cowboy Cave* [54]	*12) Gamla* [63]	

.

Considering all functional parameters: the central location of the perforation, the size and weight of the stones, their shape, raw material, the shape of the holes and their size, it seems that the perforated pebbles from NEG II are best suited to have functioned as spindle whorls. This could be strengthened with use-wear analysis, yet it is beyond the scope of the present article. Nevertheless, our initial step was to conduct a feasibility test, that resolved the uncertainties and showed that these perforated pebbles could serve to spin fibres.

#### Feasibility test

**Fig 7:**
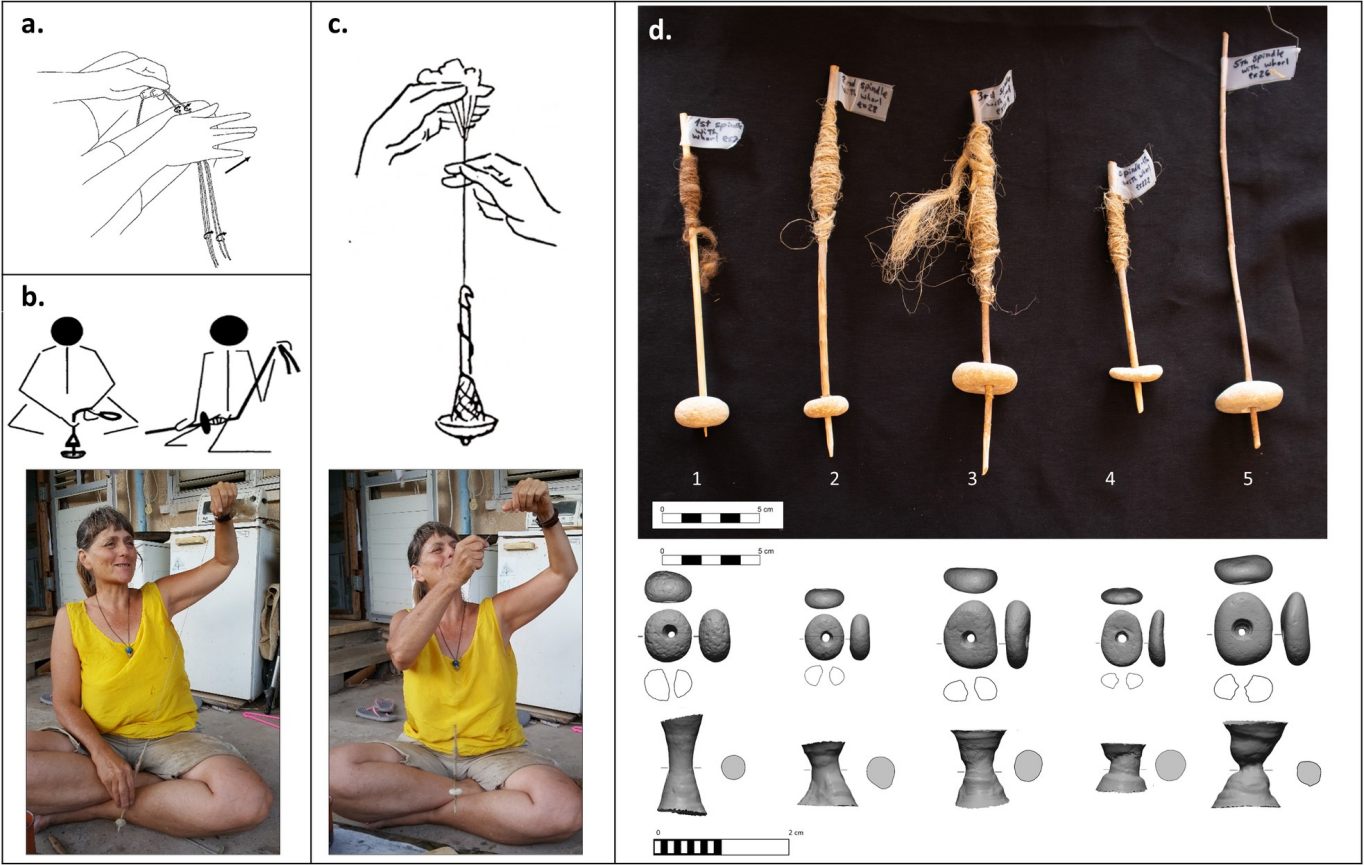
Spinning methods. (a) Manual thigh-spinning [64]; (b) Spindle-and-whorl “supported spinning” [68]; (c) “drop spinning” [66]; (d) the experimental spindles and whorls, the 3D scans of the pebbles and their negative perforations. The bottom pictures show Yonit Kristal experimenting spinning fibres with replicas of the perforated pebbles, using supported spinning and drop spinning techniques (photographed by Talia Yashuv).

Unassisted spinning, a slow spinning technology, is carried out by twisting fibres with parts of the operator’s body: between the fingers, the palm of the hand, the thigh or the toe (Fig 7A) [64, 65]. A more advanced technology, fast spinning, uses the ’spindle-and-whorl,’ where the raw fibres are tied to a wooden spindle inserted into an end-weight. When operating as a ‘supported spinning’ method (Fig 7B), the operator’s hand rotates the spindle on the ground (similar to a children’s spinning-top toy) while the other draws out unspun fibres. When operating a ‘drop-spinning’ technique, the spindle is rolled on the thigh and then dropped, continuing to rotate as it hangs in the air, and both hands are free to manage the unspun fibres [46, 66] (Fig 7C). Either way, using this implement results in a much faster and more efficient spinning process because the whorl enhances the rotation momentum of the manual twisting, meaning that the fibres continue to spin with each manual twist. It also produces a stronger and more uniform thread, with the spindle acting as a neat packaging solution for collecting the prepared thread [38, 67]

We conducted a feasibility test to further verify if the NEG II perforated pebbles could have been used as spindle whorls. The goal was to assess whether replicas of similar tools, imperfectly modified, can function for spinning fibres with the spindle-and-whorl techniques by the following protocol and criteria:

1. **The replicas:**
  - Local raw Material: similar soft limestone pebbles were collected from the Sea of Galilee Ein-Gev shore.
  - No shape modification: a similar variety of shapes were chosen without modifying their shape contour.
  - Small sized: similar small-sized pebbles were chosen, of light and medium weight.
  - Biconic perforation: holes were made with flint perforators by bi-directional manual perforation, and without widening the meeting point to imitate the biconic-shaped perforation with minimal sized width.
  - Diversity: The replicated whorls chosen for the spinning test were round, oval and irregular-shaped, of light and medium weight (4.3 g, 4.7 g, 8.8 g, 11.8 g, 15.8 g).
2. **The Expert**—we approached Ms Yonit Kristal, an acknowledged expert in traditional craft-making, to try and use our replicas of perforated stones to spin fibres. We asked her to lead the spinning attempts with her practical knowledge, and recorded her technical choices.
3. **The spindles—**We requested to maintain the relatively narrow minimum aperture of the NEG II stones by inserting thin sticks as spindles. An industrial skewer (5 mm wide) was tested as a straightened stick, yet the whorl needed to be fixed by inserting fibres into the hole, without any glue. With the other four whorls, it was found easier to use natural relatively straight branches of a nearby olive tree, 2–4 mm wide (Fig 7D). Ms Kristal pointed the edges of the sticks for easy insertion into the whorls, which got well secured without further assistance.

*The test*. The first spinning, Yonit used wool, the material she knows the best, but it did not work well, surely when compared to the modern whorls Yonit uses for her work. However, as she got a better grip and yarn started to gather around the spindle, she said that spinning, even just with this kind of whorl, is indeed faster and more efficient than spinning manually without any implements. Similar observations were reached also in other comprehensive experiments that compared thigh and finger spinning with spindle spinning [64]. In the next three tests, Yonit used flax, alternating supported and suspended spinning techniques and adjusting the effect of the whorl’s weight to the thread’s thickness. She expressed satisfaction with the pace of work.

*Results*. The current feasibility test did not control all the possible variables [40, 64, 69], as we focused only on verifying whether the morphological qualities of the NEG II perforated stones enabled them to function as spindle whorls. The experiment demonstrated that not only do the replicas function well as spindle whorls but that the parameters we suspected as disadvantageous were actually beneficial for this purpose.

Firstly, while the heavier whorl (12 g.) was the easiest for maintaining a swirl, the lighter whorls (4 g.) managed to produce thinner threads, as previous experiments established [46], and the challenge surpassed as thread piled on the spindle adding weight to the entire implement. Secondly, although biconical holes were questioned as befitting spindle whorls [45], the feasibility test also showed that the biconical rather than straight hole shape of the perforations is useful for easily setting the whorl onto the spindle. If needed, a small quantity of fibre or woodchip is enough to fix the whorl to the spindle, an action also recorded with straight holes [48]. This practice of inserting the spindles defines the perforation size and may also explain the standard minimal width of the NEG II stones.

Most importantly, we found out that perfectly round artifacts are not a prerequisite. The fact that the hole and the centre of mass are located at the item’s centre was enough for the task. The Natufian inhabitants of NEG II could have modified standard round artifacts, as exemplified by several perfectly rounded stones and the bead industry recovered on site, yet they chose not to. It is therefore suggested that the selected pebbles’ natural shapes functioned well and that the few nicely shaped items were modified due to other, perhaps aesthetic, considerations.

## Discussion

**Fig 8:**
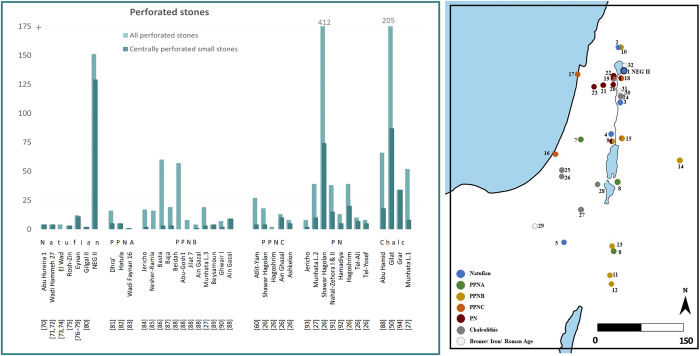
Perforated stones and spindle whorls from the Southern Levant. Left: Counts of all perforated stones, regardless of sub-typologies and morphological variations (light green) and perforated stones with similar morphological characteristics as those from NEG II (dark green): centrally perforated, small (<5 cm). Right: A map with the location of NEG II, and sites with spindle whorls or alike that are mentioned in the text and figures [27, 26, 50, 60, 70–94].

Small, centrally perforated, lightweight, un/modified stones, were also found in other archaeological sites. Accurately monitoring their distribution is a challenging task due to inconsistent documentation and different typological classifications missing, in some cases, the raw data. For example, they fall into a wide range of categories, including descriptive classifications such as ’perforation on disc,’ ’perforated discs,’ ’pebbles with central perforation,’ and ’perforated items,’ and also in functional categories such as ‘weights’ and ‘spindle whorls.’ Given these limitations, we present the assemblages of small centrally perforated stones from the Southern Levant relative to the general perforated stones (Fig 8).

**Table:** 

*1) NEG II*	*11) Baja*	*21) Tel Yosef*	*31) Tell Rehov*
*2) Eynan*	*12) Basta*	*22) Tel Ali*	*32) Gamla*
*3) Wadi Hammeh 27*	*13) Ghwair I*	*23) Nahal Zehora I*, *II*	
*4) Gilgal II*	*14) Jilat 7*	*24) Abu Hamid*	*Mentioned sites with spindle whorls but out of map scope*: *Abu-Hureira 1*, *Cowboy Cave Utah*, *EU UP roundelles*.
*5) Rosh Zin*	*15) Ain Gazal*	*25) Grar*	
*6) Dhra*	*16) Ashkelon*	*26) Gilat*	
*7) Hatula*	*17) Atlit Yam*	*27) Ashalim Cave*	
*8) Wadi Faynan 16*	*18) Sha’ar Hagolan*	*28) Qina Cave*	
*9) Jericho*	*19) Munhata*	*29) Kadesh Barnea*	
*10) Beysamoun*	*20) Hamadiya*	*30) Tell Abu Kharaz*

The NEG II perforated pebbles assemblage stands out in frequency, with only sporadic specimens from other Natufian and PPNA sites (Fig 8). As the increasing number of sites in the PPNB are mostly reported as to have dissimilar perforated stones, parallel assemblages appear only from the Pottery Neolithic, with a wide geographic distribution around the Levant [103]. From this point in time, the perforated artifacts made of stones or ceramics, mostly reported as spindle whorls, are recovered through all the periods up to historical times [104].

The absence of spindle whorls before the Neolithic led to the idea that manual spinning was used until spindle whorls started appearing in the PPNB [38], first recognized in Jericho [93]. Levy suggests an even later technological leap: “PPNB sites do not show any unequivocal evidence for spindle whorls," and therefore, "the yarn was twisted and counter twisted/doubled (spun and plied) on the thigh” (p. 63) [67]. She considers the following, Pottery Neolithic, as the “earliest culture in the southern Levant with unequivocal evidence for tool assisted, spindle-spun yarn” (p. 78) [67]. The flourishing of spindle whorls in the Late Neolithic was understood to indicate a change in spinning technology that is associated with the shift from long vegetal fibres to short animal hair spinning [38, 103].

However, while the presence of spindle whorls certainly marks the use of a fast-spinning technology, the lack of spindle whorls does not necessarily imply an unassisted manual spinning technique. In principle, this cannot be accepted in light of the range of spinning artifacts made of perishable materials known from ethnographic records. These technologies include, for example, using a spindle without a whorl, which acts as manual thigh spinning, or using a rock as a weight attached directly to the fibres without a spindle, which acts as ’drop-spinning’ [66, 105]. Additional implements, such as a wooden spindle with wooden ‘cross’ weights, a wooden spindle thickened at its end, and the use of organic whorls such as roots, coconuts or a small green potato, are also documented [47, 106, 107].

As shown earlier, during the post-Natufian period, there is a sharp decrease in the number of spindle whorls, although there is substantial evidence for fibrecraft [67]. For example, the unique comb piece from Wadi Murabba’at Cave (10k BP), which suggests that mastering the spinning process seems to have started earlier than previously thought [108, 109]. Furthermore, Bar-Yosef had stated that if this comb indeed records the presence of domesticated flax, it possibly also marks why flax domestication preceded that of edible plants and animals, suggesting it was domesticated by the Natufian culture or even earlier [110].

We conclude that the perforated stones from NEG II represent early evidence for the adoption of spinning with the “spindle and whorl” device. Following the above, we find two possible explanations for their function–either assisting fast spinning using, for the first time, these centrally perforated stone whorls replacing manual spinning, or these artifacts replaced some ’invisible/perishable’ implement of fast spinning.

The non-linear dynamics of innovation processes can be explained by the many potential points of acceptance and rejection of any new idea. Meanwhile, changes through inventions modifications are integral to this process of innovation, i.e., adoption and diffusion [111]. One factor shown to affect the successful integration of new technology is the ability to recombine existing knowledge in new ways [112–114]. Ultimately, after an innovation is adopted and firmly integrated, it becomes common knowledge. Thus, combinatorial evolution can be examined from two directions: first, regarding how an invention came into being, and second, how its components construct future technologies that evolved and developed further [115].

**Fig 9:**
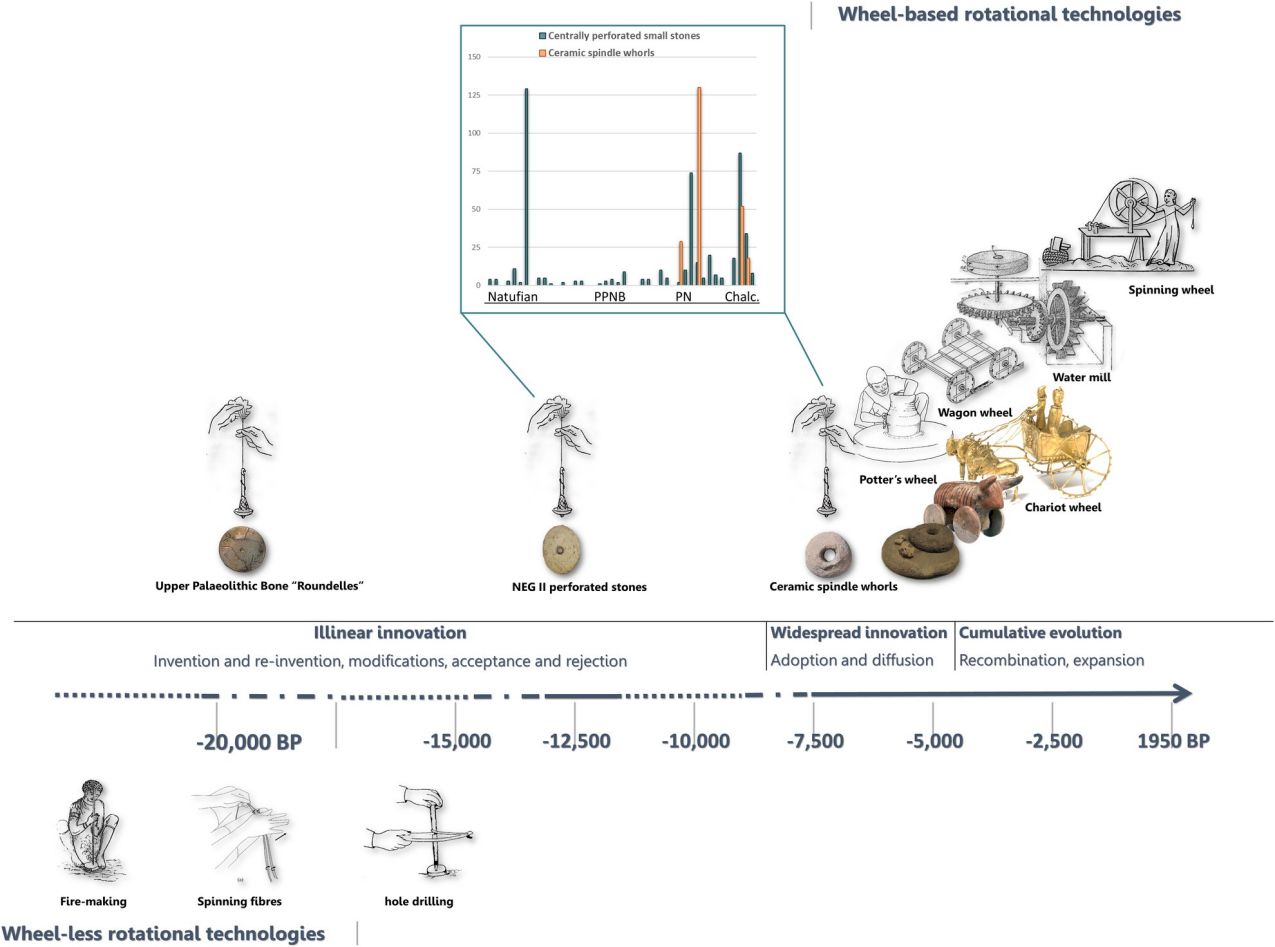
Rotational technologies: The evolution from ‘wheel-less’ to ‘wheel-based’ rotational technologies. Insert: centrally perforated small stones and ceramic spindle whorls. Figures based on: UP bone Roundelles [62]; NEG II perforated pebble photographed by Talia Yashuv; Ceramic spindle whorl [95]; Potter’s wheel [96, 97]; Wheeled animal and cart illustration [98, 99]; Golden Chariot [100]; Vitruvius water mill illustration [101]; Spinning wheel illustration [102].

In light of the above, the technological knowledge witnessed at NEG II probably did not disappear. However, it is only when spindle whorls reappear extensively in the PN that this spinning method is thoroughly assimilated. At this point in time, spinning with a spindle-and-whorl could have become common knowledge, thus creating the technological grounds for future innovations. With the notion that “any innovation in a cultural lineage is cladogenetic, creating a new branch in an evolutionary tree” (p. 12) [116], we look at how the prehistoric knowledge of one rotational technology had an impact on facilitating the evolution of additional rotation-based technologies that became essential soon after (Fig 9).

The rotating potter’s wheel was introduced during the Chalcolithic (5000–3700 CalBP), and in its initial stages, the slow ‘tournette’ was used only for the final modification stages, for a selective type of vessel [117]. With time, the use of kinetic energy onto the clay to modify it, revolutionized ceramic production when the ‘wheel-throwing technique’ was created, possibly during the Middle Bronze Age in the 2nd millennium BC [118, 119].

During the 6th millennium BP, there was a simultaneous appearance of wheeled vehicles in several regions of the Near East, the Balkan and Europe [120, 121], mostly believed to have developed from using sledges and animal draught traction for agricultural works [122–124]. As with the evolution of the potter’s wheel, wheeled vehicles were created by integrating a rotational mechanism into an existing functional form, that is, through a recombination process.

The essential mechanical element of ‘the wheel and axle’ is being capable of transforming linear to rotary motion and vice versa [2, 3], a concept that is exercised by spinning fibres as the hands move linearly while the fibres rotate and spin (see Fig 7A). The importance of using a whorl lies not only in its contribution to pacing up the spinning process itself but in integrating a circular object connected at the centre to a bar–a wheel and axle. Once the mechanical principle was routinely used in various applications, it was expanded, recombined, and incorporated with minimal modifications within other domains, which in turn resulted in a cumulative evolutional trend [9, 125] of the rotation group of technologies (Fig 9).

In the current study, we have shown how the perforated pebbles from NEG II provide evidence of a 12,000 years old wheeled-shaped tool harnessed in a rotational mechanism. We suggest, therefore, that spindle whorls, including those from NEG II, relate to the evolution of the ensuing rotational technologies by laying the mechanical principle of the wheel and axle, thus supporting the notions presented by Childe [4, 122, 126].

Material culture from NEG II in particular portrays innovations also in other domains, including the production of high-quality lime plaster [16], the presence of storage installations [17], the use of hafted flint perforators for fast drilling [18], the production of disc-beads [14], and hunting habits that show how with the diversification of task-specific activities there appeared growing specialization and possibly a greater division of labour [127]. Hence, all of those including the spindle whorls promote the idea that the inventiveness of a social group "implies that the more minds in one generation, the more novel recombinations, insights, and lucky mistakes will exist for the next generation to recombine, refine, and extend across domains” (p. 111) [128]. This notion reinforces that technological innovations are an important driving force in the Neolithization processes [129–131]. In light of the above, the innovation trends of both spindle whorls and rotational technologies provide an additional facet that reflects how Natufian innovations created realities that could not be reversed.
